# Gastrointestinal Stromal Tumor in Pregnancy: A Case Report

**DOI:** 10.1155/2009/456402

**Published:** 2009-09-16

**Authors:** S. Scherjon, W. F. Lam, H. Gelderblom, F. W. Jansen

**Affiliations:** ^1^Department of Obstetrics, Leiden University Medical Center, P.O. Box 9600 (K6-26), 2300 RC Leiden, The Netherlands; ^2^Department of Obstetrics and Gynecology, IJsselland Ziekenhuis, Capelle a/d IJssel, The Netherlands; ^3^Department of Clinical Oncology, Leiden University Medical Center, Leiden, The Netherlands; ^4^Department of Gynecology, Leiden University Medical Center, Leiden, The Netherlands

## Abstract

*Background*. Gastrointestinal stromal tumors (GISTs) are the most common mesenchymal tumors of the digestive tract and are diagnosed relatively seldom in pregnancy. *Case*. We describe a remarkable clinical course and long-term outcome, now nine years after first diagnosis, of a massive and metastatic, with a high malignancy grade GIST case, found in and treated from the first trimester of pregnancy onwards. *Conclusion*. GIST occurring during pregnancy is extremely rare. However, early diagnosis is important for optimal management. The recent better understanding of oncogenesis, the use of immunohistochemistry for differential diagnosis of GISTs, and the use of imatinib mesylate as the treatment of first choice are—as shown in this case—important for care of pregnant women with this type of malignancy.

## 1. Introduction

Gastrointestinal stromal tumors (GISTs) are the most common mesenchymal tumors of the digestive tract [1]. They most often occur in the stomach followed by the duodenum [2]. The availability of therapeutic options for metastatic GISTs [3] as well as the availability of reliable immunohistochemical staining methods has raised the reported incidence to 12.7 per million inhabitants, as published in a Dutch nation wide survey [4]. Given the age distribution of occurrence, a diagnosis of GIST during pregnancy is very uncommon [5, 6]. Those few cases reported however were symptomatic and found in the second half of the pregnancy, leading to an emergency caesarean section in one case due to fetal distress during laparotomy [5]. In the case report of Lanzafame both fetal and maternal outcome were unfortunately not reported [6]. Here we report the clinical course with long-term followup of a massive and metastatic GIST found in the first trimester of pregnancy.

## 2. Case Report 9 Years

A 25-year-old woman in her first pregnancy with an unremarkable medical and family history was referred during the first trimester to the Department of Obstetrics because on palpation the uterus was considered too large for her gestational age. She had no history of an irregular menstruation cycle. At a gestational age of 10 3/7 weeks (June 2000) the fundal height as estimated by external examination—at that gestational age just palpable at the symphysis—was already at the level of the umbilicus, comparable to a gestational age of 24 weeks, while at ultrasound the crown rump length was in accordance with the menstrual age. On routine ultrasound the uterus was found normal; the ovaries were not visualized. Dorsal of the uterus a nonspecific mass of 10 cm × 7 cm with both echodense and echolucent characteristics was seen. A more detailed ultrasound, performed at the Prenatal Diagnosis Unit making use of the highest quality ultrasound technology, was repeated a week later. Findings were similar as the week before. The mass dorsal of the uterus was unchanged. Color Doppler Sonography showed extensive vascularity within the mass. Clinically she had no complaints or any other symptoms. On physical examination the fundus was palpated in accordance with gestational age (11 3/7 weeks: 3 cm above the symphysis), however an irregular tumor, which could be palpated separate from the uterus, stretched from the umbilicus to the pelvic region could be delineated. Except for a slight-pregnancy related increase in tumormarker Cancer-Antigen 125 (CA 125: 49 nmol/L), other laboratory findings, including CarcinoEmbryonal Antigen (CEA) showed no abnormality. In normal pregnancy an elevation of CA 125 serum levels is found [7].

Because of the suspicion of an ovarian mass at a gestational age of 15 weeks and 1 day, a laparoscopy was performed. It was converted to laparotomy because of suspicion of a malignancy originating from the left ovary. However, at palpation and inspection the uterus and the both ovaries were found normal. A tumor mass of more than 20 cm diameter was located behind the uterus. The tumor was attached extensively to the jejunum, the appendix, the epiploic appendices and to the rectouterine pouch. The tumor was resected en bloc with the appendix and a part of the jejunum. As multiple small lesions were found on the spleen at inspection, which were thought macroscopically during the operation to be of possible metastatic origin, a splenectomy was performed. Because of this finding the procedure was considered nonradical.

**Macroscopic Description of the Surgical Specimens**

The large tumor was divided in 2 fragments: measuring 17 × 16 × 5 cm and 13 × 8 × 3.5 cm, respectively. Both fragments had a similar macroscopic morphology. The tumor had a yellow-gray, irregular, coarse to nodular surface. Internally there were multiple cysts with hemorrhage and central calcification. The second fragment included the appendix and a small part of the coecum, which were surrounded by the tumor. The spleen measured 9.5 × 8 × 5 cm. A few yellow-gray nodules were seen under the capsule. Internally, irregular yellow-gray nodules were found throughout the entire spleen.

**Microscopic Description**

Spindle cells orderly organized in fascicles separated by stroma were seen in both fragments of the tumorous mass, in the jejunum and in the nodules of the spleen. The cells had vague indistinct borders. The nuclei were round oval to stretched with granular chromatin. Hyaline degeneration was located in the center of the nodules. Macrophages with iron pigments were scattered over the tissue. Mitotic figures were only found in the tumor near the jejunum with a mitotic rate of 5/50 HPF.

Immunohistochemistry showed tumor cells to be positive for CD 117/c-kit, and slightly positive for MS-actin and SM-actin. The tumor cells were negative for CD 34, S 100, and only focally positive for vimentin. A final conclusion of an in part cystic degenerated GIST was made of the jejunum and metastatic nodules on the spleen.

The postoperative course was complicated by an obstructive ileus. At relaparotomy, performed 10 days after the initial operation, a tumor was palpated in the horizontal part of the duodenum. Gastroscopy during the procedure revealed a polypoid tumor in the distal is duodenum obliterating the lumen. Duodenal biopsies and lymph node samples were taken. As the type of surgical interventions is depending on the histology of the tumor it was decided to wait for the results of the histopathologic examinations of the biopsies before additional intervention was performed.

At *pathological investigation* the biopsies and the abdominal lymphnodes consisted of spindle cells similar to findings in the tumor and in the spleen and were diagnosed as GIST localizations rather than lymph nodes. Five days after the second operation, when the definitive histopathology was known at a gestational age of 17 weeks and 2 days, a third—now palliative procedure was performed. The horizontal part of the duodenum was resected over 15 cm and a gastroenterostomy was made Macroscopically, at the stenosis there were signs of hemorrhage, necrosis, and inflammation in the submucosa. No tumor was seen. Microscopically, a GIST localisation of 2 mm was seen in the submucosa. 

After the third operation patient's condition improved rapidly. Pregnancy was uncomplicated until week 41 of gestation, when she developed a mild pregnancy induced hypertension. No antihypertensive treatment was started. At 41 weeks and 6 days labour started spontaneously. She delivered vaginally of a healthy boy, with a weight of 3990 gr. (75 percentile) and an Apgar score 9/10 after 1 and 5 minutes, respectively. The next day the woman and child could return home in good condition. In a multidisciplinary meeting held after the delivery it was concluded that no further medical options would possibly improve maternal outcome. She had not been enrolled in the phase III imatinib trial [8], open for entry at the time of diagnosis, due to the absence of measurable disease after resection of all visible tumor.

Three years after the initial diagnosis she was pregnant for the second time. Both pregnancy and delivery were uneventful. At five years followup she was still in complete remission. On magnetic resonance imaging, repeatedly performed during this period, no recurrent tumor nor distant metastases were found. However, one year later, at a follow-up CT scan, a solid lesion was seen in front of the horizontal part of the duodenum, with a diameter of 8 cm. At laparotomy the tumor with a diameter of 11 cm was removed, but also possible tumor deposition was seen on the right ovary and the peritoneum. The tumor was composed of spindle like cells. The mitotic frequency was 20/50 HPF. Immunohistochemistry was comparable with a GIST localization. Microscopy and immunohistochemistry of lesions suggestive for peritoneal metastasis and metastasis on the right ovary were also GIST tumor cells. Sequence analysis showed the typical mutation in Kit exon 11: W557R (TGG → CGC).

The right ovary was removed 2 months later and also biopsies were taken from possible tumor residues on the left ovary. Five small localizations with a maximal diameter of 0.8 cm composed of spindle like cells were, with a mitosis frequency of 20 per 50 high power field (HPF), suggestive of GIST lesions found in the right ovary. Immunohistochemical findings are comparable to the findings in the primary tumor and to the metastic tissue removed during the fourth operation. CD117 was strong positive, vimentine and CD34 were positive, and MSA and MS-actine were weak positive. 

Two months later, 6 years after the first surgical procedure, again a paramedian disease recurrence was found by CT scan. It was now decided to start with imatinib therapy (400 mg 1 tablet/day) because of recurrent metastatic disease. At follow-up CT scans performed 1/2, 1 and 2 years after starting the imatinib therapy no tumor residue could be demonstrated (complete radiological response). However, at the CT scan two cystic lesions were seen at the left ovary, which were removed in March 2008 during a laparoscopic partial ovariectomy. The cysts turned out to be a corpus luteum cyst. Using immunohistochemistry no localization of GIST could be demonstrated. No other macroscopic tumor localizations were seen during laparoscopy.

## 3. Discussion

This case report of a tumorous process diagnosed during pregnancies stresses the importance of a long-term multidisciplinary approach between different specialities, including obstetrics, surgery, pathology, imaging, and medical oncology in the treatment of a GIST during pregnancy. It also shows the course of a long-term survival—even of a metastatic GIST considered to be of high risk—in good quality of life. GIST tumours are seldom, especially at younger ages. This explains the limited experience with these tumours diagnosed during pregnancy [6]. In this limited experience no reference is made to the possibility of metastatic disease in the fetus or the developing of GIST in utero. Early diagnosis and the start of optimal management treatment are supposed to be of importance [3], although our case is illustrating that even with positive tumour margins and a relative late start of imatinib long-term survival—more than nine years after initial—disease is possible. Imatinib was started only when evaluable metastatic disease occurred, as adjuvant treatment after (even complete) resection is considered experimental and should be evaluated in ongoing trials [3]. Our patient was not included in a trail because she was pregnant. 

Gastointestinal stromal tumor (GIST) is the most common abdominal mesenchymal tumor with an incidence of approximately 12–14 per million [4, 9]. The incidence of GIST with a clinical malignant behaviour is estimated 45% of the total incidence [4]. Compared to other gastrointestinal tumors such as colon carcinoma with an incidence of 30/100 000 or gastric carcinoma with an incidence of 16/100 000 GIST is a relatively rare neoplasm. GISTs contribute 2.2% to gastric cancers, 13.9% to small bowel cancers, and 0.1% to colorectal cancers [10]. GISTs generally occur in adults, with a median range between 55 and 65 years [1, 2]. There are probably no sex differences in occurrence [2]. The increased interest on GIST and the consensus on the diagnostic criteria of GIST has raised the incidence of the disease [4].

GISTs can develop in the entire gastrointestinal tract and occasionally in the omentum and mesentery. The stomach (60–75%) and the small intestine (20–30%) are the most common localizations of the tumor. GISTs in the esophagus and colon are relatively more frequently showing a malignant behavior [1, 2]. GIST may spread intra-abdominally to the omentum and peritoneum or reoccur locally after nonradical resection of the primary tumor. Metastasis can be found in liver and only very rarely in lungs, bones and subcutis [1, 2]. The spleen involvement as seen in our patient is a rare finding [11]. The size of the GISTs may vary from small (1-2 cm) to more than 20 cm, as described in our patient [2]. The clinical presentation, such as pain, nausea, vomiting, bleeding, obstruction, anaemia, melaena depends on the tumor size and localization. The presentation is nonspecific and is therefore of limited use for diagnostic consideration [2]. A significant part of GISTs remains asymptomatic and is detected incidentally [12]. This is clearly illustrated in our case, as the patient was free of any symptom, despite the considerable tumor size and multiple localizations.

In the recent years GIST has been recognized as a distinct entity, apart from leiomyomas, leiomyoblastomas or leiomyosarcomas. GIST has been defined as mesenchymal tumors of the gastrointestinal tract composed of spindle, or epithelioid, or occasionally pleomorphic cells that frequently express the c-kit protein, as detected using immunohistochemistry [1]. Immunohistochemical determination has become the corner stone in the differential diagnosis of mesenchymal tumors. The c-kit protein (CD117), a growth factor receptor for stem cell factor, is positive in approximately 85–90% of cases. CD34, a hematopoietic progenitor cell marker normally present on vascular endothelium and a subset of fibroblast, is positive in 60–70% of GISTs. Smooth muscle actin (SMA) and muscle specific actin (MSA) are expressed in smooth muscle and some myofibroblasts. In GISTs muscle actins may be present in 20–40% of the cases. SMA is often reciprocal with CD34 expression. In GISTs S-100 is positive in 10% of the cases. Heavy caldesmon (HCD), an actin binding cytoskeleton associated protein, which is normally present on smooth muscle and myoepithelial cells, is generally positive in GISTs and smooth muscle tumors. Vimentin is also generally positive in GISTs [1, 10].

There are no uniform criteria to predict the biological behavior of GIST. The mitotic rates in combination with tumor size and tumor site [13] seem to be the most important prognostic factors. Fletcher has proposed a risk assessment dividing into four categories:

1. very low risk (<2 cm and <5/50 HPF);
2. low risk (2–5 cm and <5/50 HPF);
3. intermediate risk ((a) <5 cm and 6–10/50 HPF or (b) 5–10 cm and <5/50 HPF);
4. high risk ((a) >5 cm and >5/50 HPF or (b) >10 cm and any mitotic rate or (c) any size and >10/50 HPF) [10].

Localization may also influence the prognosis. The small intestinal GIST is associated with the poorest long-term survival rate, whereas patients with stomach GIST had the best survival. 

In the recent years rapid progress has been made on the understanding of the oncogenesis of GISTs. The gain-of-function mutation in the c-kit proto-oncogene, which can be found in 90% of GISTs, seems to be the basis of the pathogenesis. This genetic aberration leads to an unbridled stimulation of c-kit receptor and overexpression of the tyrosine kinase protein and a subsequent growth and antiapoptotic behavior of tumor cells [14, 15]. The introduction of the receptor tyrosine kinase inhibitor STI-571 (imatinib mesylate or Glivec/Gleevec) heralds a new era in the treatment of GIST. Promising results have been reported in clinical trials on the metastatic disease [8, 16]. Until recently surgery was the only successful treatment of GISTs. Chemotherapy and radiotherapy had proven to be ineffective. Therefore, only localized disease could be curatively treated. Because in our case imatinib was not applicable because of the pregnancy she has been primarily been operated. 

Our case had a remarkable clinical course. Despite metastatic GIST and the extensive surgical interventions in the second trimester, the pregnancy had developed uneventfully. Furthermore, she was in complete remission in the five-year followup. This is highly unexpected in view of the unfavorable prognosis due to the considerable size (20 cm) and localization of the primary tumor (jejunum), the high mitotic rate (20/50 HPF), and the metastatic presentation (peritoneum) of the GIST. According to the classification by Fletcher she has to be classified as a high risk patient (>10 cm and any mitotic rate) [10].

Because she had no measurable disease as evaluated by repeated CT scans she was not treated with imatinib after the delivery. Also it is not clear whether imatinib therapy started early in the treatment course is more effective than at with the moment of more advanced disease it was decided not to start with this medication [17]. With recurrence of disease—more than 6 years after surgery of the primary tumor—the start of this medication had a strong effect on the locoregional metastasis as demonstrated by a complete response at CT scan and confirmed by laparoscopy more than one year after start of treatment. 
